# An Efficient and Stable Registration Framework for Large Point Clouds at Two Different Moments

**DOI:** 10.3390/s24227174

**Published:** 2024-11-08

**Authors:** Guangxin Zhao, Jinlong Li, Jingyi Xi, Lin Luo

**Affiliations:** 1 School of Optoelectronic Science and Engineering, University of Electronic Science and Technology of China, Chengdu 611731, China; zhaoguangxin123@outlook.com; 2 School of Physical Science and Technology, Southwest Jiaotong University, Chengdu 611756, China; xijingy9@163.com (J.X.); happyluolin@vip.163.com (L.L.)

**Keywords:** cloud point, registration, large scale, random sampling, train components

## Abstract

Point cloud registration plays a great role in many application scenarios; however, the registration of large-scale point clouds for actual different moments suffers from the problems of low efficiency, low accuracy, and a lack of stability. In this paper, we propose a registration framework for large-scale point clouds at different moments, which firstly downsamples large-scale point clouds using a random sampling method, then performs a random expansion strategy to make up for the loss of information caused by the random sampling, then completes the first registration by a deep learning network based on the extraction of keypoints and feature descriptors in combination with RANSAC, and finally completes the registration using the point-to-point ICP method. We conducted validation experiments and application experiments on large-scale point clouds of key train components, and the experimental results are much higher in accuracy or efficiency than other methods, which proves the effectiveness of our framework, which can be applied to actual large-scale point clouds.

## 1. Introduction

With the constant development of technology in the fields of industry and agriculture, biomedicine, transport and so on, the processing and analysis of 3D point clouds has become an important task. Due to the influence of various external factors during data acquisition, the point clouds of the same target will have missing information and differences in the acquisition process, which are reflected in their shape, position and other aspects. Among the traditional registration methods, the iterative closest point (ICP) algorithm [1] is the most widely used, which can ensure the accuracy, convergence speed, and stability of registration under ideal conditions. Traditional feature-based algorithms are represented by point feature histograms (PFH) [2] and fast point feature histograms (FPFH) [3], which match feature points to achieve registration by finding the feature information of two slices of point clouds. Traditional registration methods have characteristics of stability, but their registration efficiency is low and time-consuming, while deep learning methods can perform registration quickly and efficiently. In recent years, deep learning methods have performed well on public datasets, but for actual large-scale point cloud datasets, deep learning methods are unstable, making them difficult to be applied.

Huang et al. [4] summarized deep learning-based point clouds registration algorithms, which can be classified into two categories: end-to-end learning algorithms and feature learning-based algorithms. End-to-end learning algorithms are specifically targeted at designing end-to-end neural networks for registration problems, and the estimation of the transformation matrix is added to the optimization of the network so that it can directly predict the transformation matrix of the two input point clouds. Qi et al. pioneered a deep neural network, PointNet [5], which can be directly input to point clouds and can efficiently extract the global features of the point clouds. PointNetLK [6] combines PointNet with the traditional image registration algorithm LK to construct a recursive neural network, which estimates the transformation matrix by minimizing the feature difference between two features, which has a high efficiency and ensures registration accuracy. Huang et al. [7] further improved PointNetLK by using an autoencoder method and point distance loss.

Feature learning-based algorithms are more widely used currently, which regard deep neural networks as a tool for extracting features, focusing on learning point features, estimating accurate correspondences, and then completing the prediction of the transformation matrix through singular value decomposition (SVD) [8] or RANSAC [9] one-step optimization. 3DMatch [10] is one of the representatives, which extracts the local geometric features of the point cloud based on deep learning to complete the matching of keypoints. The point-to-point feature network (PPFNet) [11] uses PointNet as a feature encoder to extract robust local feature descriptors, and PPF-FoldNet [12] improves on this by incorporating FoldingNet [13] to make the features rotationally invariant. Unlike PointNet, the dynamic graph convolutional neural network (DGCNN) [14] combines the graph convolution idea and proposes the EdgeConv module, which can participate in the network design as a base module for extracting the global and local features of the point cloud. DCP [15] is a typical algorithm using DGCNN for feature extraction, and the algorithm also introduces a transformer [16] to adjust the features, which can better estimate the relationship between two slices of point clouds. However, DCP performs poorly on partial registration. Wang et al. proposed a partial registration network, PRNet [17], to optimize for this problem. The RGM [18] framework draws on the DCP architecture and proposed for the first time the use of depth map matching to solve the point cloud registration problem. USIP [19] focuses on solving the problem of keypoints and feature extraction for point clouds.

Recently, Wu et al. [20] proposed a multiform approach to solve the point cloud registration problem through evolutionary multitasking, which demonstrated powerful capabilities. For the partial overlap registration problem, StrucTure OveRlap Match (STORM) [21] designs an overlap prediction module with differentiable sampling, which achieves accurate partial correspondence generation. Wu et al. propose an inliers estimation network (INENet) [22] to extract overlapping regions and use high-overlap registration methods to match overlapping regions, which transforms the partial overlap registration problem into a problem of extracting overlapping regions. Meanwhile, Wu et al. proposed a skip attention-based correspondence filtering network (SACF-Net) [23] to enable the decoder to extract high-quality correspondences within the overlapping regions. The partial-to-partial registration network (RORNet) [24] selects a small number of keypoints from the estimated overlaps, called reliable overlap representations, thus reducing the side effects of overlap estimation errors on registration. The point attention-based multi-scale feature fusion network (PANet) [25] uses multiple branches to extract local features at different scales in parallel before fusing them to enhance the representation of the features, which improves the accuracy of registration more than fixed-scale local features.

3D large-scale registration is less studied in previous works. Based on the idea of random sample consensus [9] (RANSAC), Aiger et al. proposed 4-points congruent sets (4PCS) [26], which constructs a coplanar four-point set from source and target point clouds and utilizes the approximation of the point set under rigid transformation to consistently obtain eligible matching point pairs and use the maximum common point set as the metric to find the maximum overlap four-point pairs after registration to complete the solution of the transformation matrix. However, 4PCS has quadratic time complexity in terms of the number of data points, which greatly limits its applicability for acquisition in large environments. Mellado et al. proposed Super 4PCS [27], which is simple, memory-efficient, and fast, using only indexes. Mohamad et al. proposed the Super Generalized 4PCS algorithm [28], which reduces the number of congruent four-point bases through a generalized four-point base, which is more efficient than the Super 4PCS acceleration up to 6.5 times. In response to the problem that the proportion of the original point cloud is not reduced in Super 4PCS, Li et al. proposed Super Edge 4PCS [29], which improves the speed and accuracy. Pingi et al. presented a fast and simple algorithm for the automatic registration of a large number of range maps [30], which exploits a compact and GPU-friendly descriptor. Palma et al. proposed a non-rigid registration method for large 3D meshes from multi-view stereo (MVS) reconstruction [31]. Zhang et al. addressed the fact that most of the current registration methods are limited to small-scale 3D point clouds (about 4 k points) and proposed the DDRNet network [32], which makes it possible to handle large-scale scene points. RSKDD-Net [33] improves USIP, which uses random sampling to extract candidate points and solves the problem of information loss brought by random sampling by a random expansion clustering strategy. To address the problem that most existing registration methods are highly dependent on well-designed descriptors and post-processing choices, RegFormer is proposed for large-scale point cloud registration without any further post-processing [34].

**Figure 1:**
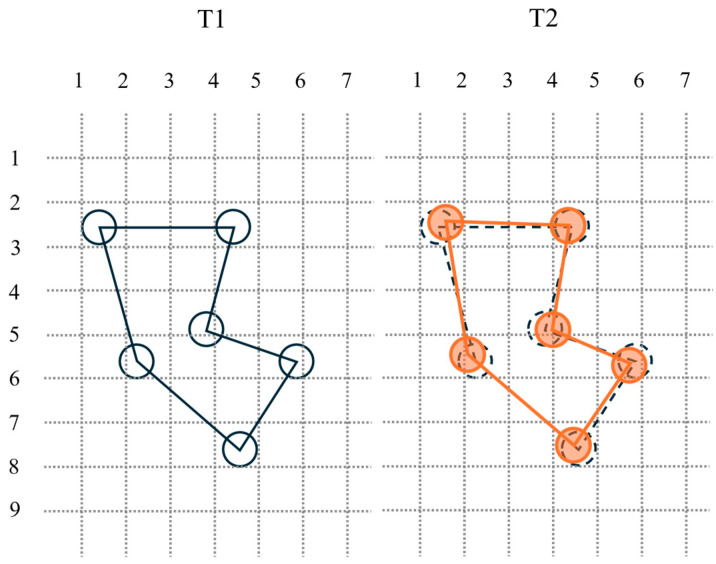
The point cloud obtained from scanning at different moments. Even without rotation and translation, the points at moments T1 and T2 cannot coincide exactly, which means there is an error in it.

However, there are still some unsolved problems in the registration of large-scale point cloud datasets. Firstly, when using traditional methods for large-scale point cloud registration, it is time-consuming and inefficient and sometimes even fails to register and cannot meet the requirements of practical applications. Deep learning methods will greatly improve the registration efficiency, reduce the time, and improve the registration accuracy, but at the expense of stability, which means that the results are highly dependent on the datasets and the training process, i.e., the registration results obtained from different point clouds after training and testing may be very different, and it is difficult to meet the requirements of stability in practical applications. In addition, the existing point cloud dataset is usually a pair of point cloud pairs obtained by random translation and rotation so that such cloud point pairs are exactly one-to-one in relative positions. However, in the actual point cloud pairs obtained by scanning the same object at different times, although there is no rotation and translation, the relative positions of the points in the point cloud pairs do not exactly have a one-to-one correspondence due to the fact that the specific points scanned during each scanning process will be slightly different. Although the corresponding points can be found by the nearest neighbor method, there still exists an error, which can not be ignored for the point cloud registration. As Figure 1 shows the error, assuming that the object being scanned has no rotation and translation, the black solid line in the left figure represents the point cloud obtained from scanning at the moment of T1, the orange solid line in the right figure represents the point cloud obtained from scanning at the moment of T2, and the black dotted line is the position of the point cloud at the moment of T1. It can be seen that the point cloud pair obtained from actual scanning can not be matched one-by-one in relative position, which means there exists an error.

Aiming at the existing problems, this paper proposes a new efficient and stable registration framework that combines a neural network based on keypoints and descriptors and the traditional fine registration algorithm ICP [1], which can be used for the rigid registration of large-scale 3D point clouds. The framework targets large-scale point cloud registration and utilizes random sampling for fast and efficient downsampling so that the point clouds enter the network smoothly. The keypoints and feature descriptors of the source and target point clouds are fully learned through the network, and the initial registration of the two point clouds is accomplished based on this point feature information by RANSAC [9] so that the positions of the point clouds are roughly registered. Finally, the ICP algorithm is used to complete the fine registration with a small amount of iterative optimization to enhance the stability of the whole algorithm. In addition, this paper designs a distance error metric to compare and measure the registration accuracy of the algorithms, which provides assistance in objectively evaluating the registration performance when the point cloud pair transformation matrix is unknown. In this paper, the effectiveness of the framework is verified on point clouds of different scales, which are from a few hundreds of thousands of points to about two million, and it demonstrates its high performance, and a large-scale point cloud dataset of key components of a train is constructed, focusing on researching and testing the practical application level of the framework, which reflects its application value.

The remainder of the paper is organized as follows. Section 2 describes the specific structure and loss function of the framework. In Section 3, we introduce the composition of the dataset, and we compare the results of different algorithms on the completed dataset to verify the validity of the framework. In addition, we complete and analyze partially overlapping registration experiments, experiments with the same and different scenario applications. In Section 4, we summarize the article and provide some concluding remarks.

## 2. Methods

### 2.1. Registration Framework

**Figure 2:**
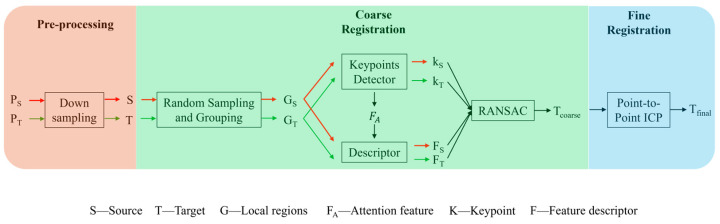
Registration framework. This registration framework consists of the pre-processing process, coarse registration process, and fine registration process. The coarse registration consists of random sampling, grouping, a keypoints detector, a feature descriptor, and RANSAC, while the fine registration is performed by the point-to-point ICP algorithm.

In this paper, we propose a new efficient and stable registration framework that combines traditional algorithms with deep learning algorithms. As shown in Figure 2, the inputs are target and source point clouds, which are first downsampled in the preprocessing stage. In the coarse registration stage, random sampling is used to efficiently sample the input target and source point clouds with large scales, followed by clustering, keypoint and feature descriptor extraction, and RANSAC matching; the coarse registration-transformed matrix $T_{coarse}$ is outputted at this stage. In the fine registration stage, the ICP algorithm is used to achieve accurate point cloud registration and the output final registration-transformed matrix $T_{final}$ after a small number of iterations to enhance the stability of the algorithm.

### 2.2. Point Cloud Registration Network Based on Keypoints and Descriptors

**Figure 3:**
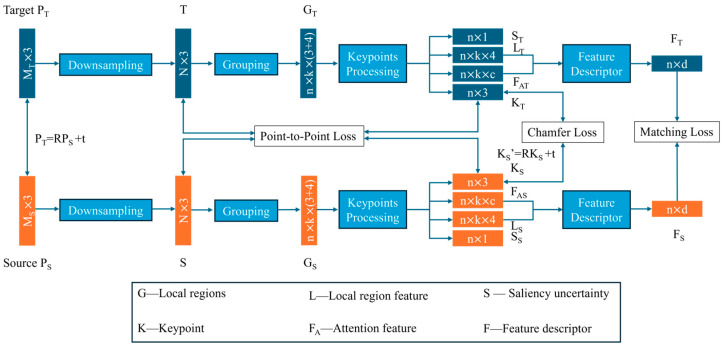
Registration network based on keypoints and descriptors. The network contains random sampling, grouping, keypoints processing, and feature descriptors, and gives the size of the data obtained at each step as well as the composition of the loss function.

As shown in Figure 3, the network uses feature learning-based registration, which is used as a tool to extract the features of point clouds and accurately obtain the keypoints and feature descriptors of the point clouds. The network consists of four parts: random sampling, clustering, keypoint processing, and feature descriptor extraction. Firstly, random sampling is used in the sampling stage to achieve the efficient downsampling of large-scale point clouds so that the point clouds are input into the network smoothly. Then, the candidate points are randomly selected, clustering is completed by finding the neighboring points around the candidate points, and the local area point sets are constructed. In the keypoint processing module, randomly selected candidate points are adjusted according to the attention weights. And then, the local point set features, the global point set features, and the attention features are combined to extract useful feature descriptors in the descriptor extraction module, which provides accurate feature descriptors for subsequent registration.

### 2.3. Random Sampling

Large-scale point clouds usually have millions of points, which makes it difficult to directly input the original point clouds into the network, so it is necessary to downsample the point clouds first. RandLA-Net [35] mentions that the advantages of random sampling are its high computational efficiency, its low computational complexity, that it does not require additional memory for computation, and that it can control the number of sampling points by itself, which make it the most suitable method for sampling large-scale point clouds. In this paper, random sampling is used to make the original point clouds sparse, which greatly reduces the point cloud density. However, random sampling may cause many useful point features to be discarded; to overcome this problem, a clustering module is added after random sampling to retain more point features.

### 2.4. Random Dilation Cluster Strategy

**Figure 4:**
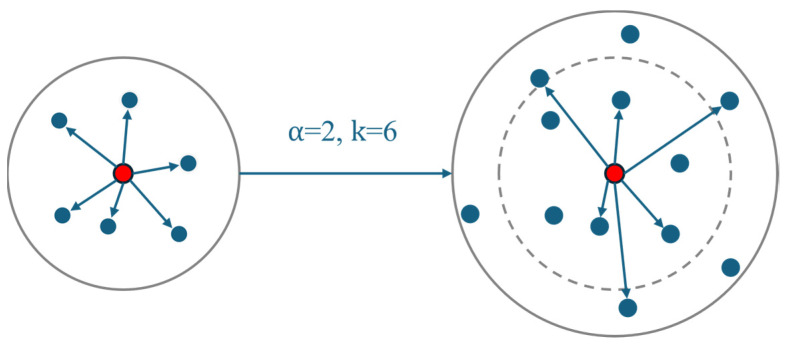
Standard k NN-based cluster (**left**) and random dilation cluster (**right**). The random dilation cluster selects α×k proximity points near the candidate point and then randomly selects k proximity points to complete the clustering.

A k-nearest-neighbor cluster will first randomly select n candidate points based on the N point clouds obtained from the random sampling in the previous stage and then select the k nearest field points, each centered on each candidate point to complete the clustering, which searches for correspondences by constructing such a keypoint set. However, since random sampling may lead to information loss, this paper uses a random dilation cluster strategy to expand the sensory field and reduce the negative impact of random sampling without increasing the number of neighborhood points. The expansion coefficient α of the method (α = 2 in this paper) is used to select neighborhood points firstly when selecting α × k neighborhood points near each candidate point and then when randomly selecting k field points from α × k neighborhood points to form a local neighborhood point set. Figure 4 shows the cluster of a single candidate point with an expansion coefficient α of 2 and neighborhood points k of 6, with a normal cluster on the left and a random dilation cluster on the right. The input point cloud for the module is an N × 3 point set and the output is an n × k × (3 + 4) point set G, where n × k × 3 contains the 3D coordinate information of each neighborhood point and n × k × 4 contains the Euclidean distances and 3D vectors between the candidate points and the neighborhood points.

### 2.5. Keypoints Processing

The main purpose of keypoints processing is to adjust the spatial position of each candidate point using the neighborhood point information. This module takes the local region point set G as an input and denotes the *i*-th candidate point and its *k* domain points as $p^{i}$ and $\left\{p_{1}^{i},p_{2}^{i},\ldots ,p_{k}^{i}\right\}$, respectively. By convolving the spatial feature information $\left\{f_{1}^{i},f_{2}^{i},\ldots ,f_{k}^{i}\right\}$, a feature $\left\{{\tilde{f}}_{1}^{i},{\tilde{f}}_{2}^{i},\ldots ,{\tilde{f}}_{k}^{i}\right\}$ can be obtained, and it can obtain the one-dimensional attention weight $\left\{\omega_{1}^{i},\omega_{2}^{i},\ldots ,\omega_{k}^{i}\right\}$ of each neighborhood point by the maximum pooling layer and the softmax function. It assigns the corresponding weight to the coordinate information of each neighborhood point $\left\{x_{1}^{i},x_{2}^{i},\ldots ,x_{k}^{i}\right\}$ to adjust the candidate points. It thus obtains the keypoint ${\hat{x}}^{i}$, the adjusted *i*-th keypoint, which can be expressed by the following equation,

(1) $${\hat{x}}^{i}=\sum_{j=1}^{k}\omega_{k}^{i}\cdot x_{k}^{i},i=1,2,\ldots ,n\;$$

The keypoints obtained after adjusting by the attention weight ensure that the generated keypoints lie within the convex hull of the input clusters. Meanwhile, this attention weight is applied to the features $\left\{{\tilde{f}}_{1}^{i},{\tilde{f}}_{2}^{i},\ldots ,{\tilde{f}}_{k}^{i}\right\}$ to obtain an attention feature $F_{A}$, denoted as

(2) $${\left(F_{A}\right)}_{j}^{i}=\omega_{j}^{i}\cdot {\tilde{f}}_{j}^{i},i=1,2,\ldots ,n;j=1,2,\ldots ,k\;$$

On this base, summing the attention features can obtain the global features of each point set, and then the significant uncertainty of each keypoint can be obtained by a multilayer perceptron (MLP). The significant uncertainty can reflect the reliability of the keypoint. After sufficient network learning and optimization, the keypoints with small significant uncertainties can be output.

### 2.6. Feature Descriptor

The function of the feature descriptor module is mainly implemented by convolution. The inputs are the local area point set features and attention features. In order to obtain more stable features for each keypoint, on the one hand, a single local feature of all its neighboring points is obtained through a two-dimensional convolution operation, and on the other hand, the corresponding global feature of the point set is obtained through two-dimensional convolution and maximum pooling. The high-dimensional features are extracted by convolution and maximum pooling after fusing the local features and global features with the attention features, and the feature descriptor F of the size n × d is finally generated, and its dimension is d.

### 2.7. Loss Function

According to USIP [19] and RSKDD-Net [33], the loss function has three components: point-to-point loss $L_{point-to-point}$, probabilistic chamfer loss $L_{chamfer}$, and matching loss $L_{matching}$. The total loss function $L_{total}$ is

(3) $$L_{total}=L_{point-to-point}+L_{chamfer}+L_{matching.}$$

- Point-to-point loss

Point-to-point loss is used to constrain the distance between a keypoint and an input point cloud to ensure that it is not too far away and to avoid the keypoint being too far away from the input point cloud. Assuming that the two pieces of point cloud input into the network are *X* and *Y*, the point-to-point loss $L_{point-to-point}$ can be expressed as

(4) $$L_{point-to-point}=\sum_{i=1}^{n}\underset{X_{j}\in X}{\min}{\left\|{\left(K_{X}\right)}_{i}-X_{j}\right\|}_{2}^{2}+\sum_{i=1}^{n}\underset{Y_{j}\in Y}{\min}{\left\|{\left(K_{Y}\right)}_{i}-Y_{j}\right\|}_{2}^{2},$$

where $K_{X}$ and $K_{Y}$ are the keypoints of the two pieces of point cloud and *n* is the number of keypoints.

- Probabilistic chamfer loss

Probabilistic chamfer loss is used to minimize the Euclidean distance between keypoints in source and target point clouds transformed by the predicted matrix.

The transform equation $P_{X}=RP_{Y}+t$ can be derived from the source and target point clouds $P_{Y}$ and $P_{X}$, which can derive that the keypoint-transformed source point cloud is $K_{Y}\prime =RK_{Y}+t$. Chamfer loss is calculated between the transformed source point cloud keypoints $K_{Y}\prime =\left\{{\left(K_{Y}\prime \right)}_{j}|j=1,2,\ldots ,k\right\}$ and the target point cloud keypoints $K_{X}=\left\{{\left(K_{X}\right)}_{i}|i=1,2,\ldots ,k\right\}$ and can be expressed as the distance

(5) $$L_{chamfer}=\sum_{i=1}^{n}\underset{{\left(K_{Y}\prime \right)}_{j}\in K_{Y}\prime }{\min}{\left\|{\left(K_{X}\right)}_{i}-{\left(K_{Y}\prime \right)}_{j}\right\|}_{2}^{2}+\sum_{j=1}^{n}\underset{{\left(K_{X}\right)}_{i}\in K_{X}}{\min}{\left\|{\left(K_{X}\right)}_{i}-{\left(K_{Y}\prime \right)}_{j}\right\|}_{2}^{2}.$$

However, the significant uncertainty *σ* is different between different pairs of keypoints, and probability chamfer loss can therefore be optimized as

(6) $$L_{chamfer}=\sum_{i=1}^{n}-\ln p\left(d_{ij}|\sigma_{ij}\right)+\sum_{j=1}^{n}-\ln p\left(d_{ij}|\sigma_{ji}\right)\;\;\;\;\;\;\;\;\;\;\;\;\;\;\;\;=\sum_{i=1}^{n}\left(\ln\sigma_{ij}+\frac{d_{ij}}{\sigma_{ij}}\right)+\sum_{j=1}^{n}\left(\ln\sigma_{ji}+\frac{d_{ji}}{\sigma_{ji}}\right),$$

where $d_{ij}$ denotes the distance between the *i*-th point ${\left(K_{X}\right)}_{i}$ in $K_{X}$ and its nearest neighbor ${\left(K_{Y}\prime \right)}_{j}$ found in $K_{Y}\prime$, and $\sigma_{ij}$ denotes the mean value of the significant uncertainty of these two points; $d_{ji}$ denotes the distance between the *j*-th point ${\left(K_{Y}\prime \right)}_{j}$ in $K_{Y}\prime$ and its nearest neighbor ${\left(K_{X}\right)}_{i}$ found in $K_{X}$, and $\sigma_{ji}$ denotes the mean value of the significant uncertainty of these two points. The specific expressions are

(7) $$\left\{\begin{array}{c} d_{ij}=\underset{{\left(K_{Y}\prime \right)}_{j}\in K_{Y}\prime }{\min}{\left\|{\left(K_{X}\right)}_{i}-{\left(K_{Y}\prime \right)}_{j}\right\|}_{2}\\ d_{ji}=\underset{{\left(K_{X}\right)}_{i}\in K_{X}}{\min}{\left\|{\left(K_{X}\right)}_{i}-{\left(K_{Y}\prime \right)}_{j}\right\|}_{2} \end{array}\right.,$$

(8) $$\left\{\begin{array}{c} \sigma_{ij}=\frac{{\left(\sigma_{X}\right)}_{i}+{\left(\sigma_{Y}\right)}_{j}}{2}\\ \sigma_{ji}=\frac{{\left(\sigma_{Y}\right)}_{j}+{\left(\sigma_{X}\right)}_{i}}{2} \end{array}\right..\;$$

Since the point clouds are oriented differently when finding the nearest neighbors, the pairs of nearest neighbors in the two point clouds are different, which means $d_{ij}\neq d_{ji}$ and $\sigma_{ij}\neq \sigma_{ji}$.

- Matching loss

Matching loss is used to train feature descriptors to minimize the distance between the descriptors of two pieces of a point cloud and bring the matched descriptors closer together. The distance between the source point cloud and each keypoint descriptor in the target point cloud is calculated. To take the feature descriptor ${{(F}_{Y})}_{j}$ of the *j*-th keypoint ${\left(K_{Y}\right)}_{j}$ of the source point cloud as an example, the set of distances between it and the keypoint $F_{X}$ of the target cloud, ${{(D}_{Y})}_{j}$, can be expressed as

(9) $${{(D}_{Y})}_{j}=\left\{{{(D}_{Y})}_{j1},\ldots ,{{(D}_{Y})}_{ji},\ldots ,{{(D}_{Y})}_{jn}\right\}\;=\left\{{\left\|{\left(F_{Y}\right)}_{j}-{\left(F_{X}\right)}_{1}\right\|}_{2},\ldots ,{\left\|{\left(F_{Y}\right)}_{j}-{\left(F_{X}\right)}_{i}\right\|}_{2},\ldots ,{\left\|{\left(F_{Y}\right)}_{j}-{\left(F_{X}\right)}_{n}\right\|}_{2}\right\}.$$

Then, the set of matching scores can be obtained by Equation (10),

(10) $${\left(s_{Y}\right)}_{ji}=\frac{e^{\frac{1/{{(D}_{Y})}_{ji}}{t}}}{\sum_{j=1}^{n}e^{\frac{1/{{(D}_{Y})}_{ji}}{t}}}.\;$$

where *t* is the parameter used to sharpen the matching score distribution, and the target point cloud keypoints with more similar descriptors to the source point cloud keypoints will obtain larger matching scores. The weighted summation of this set of matching scores ${\left(s_{Y}\right)}_{ji}$ with the target point cloud keypoints $K_{X}$ adjusts ${\left(K_{Y}\right)}_{j}$ to

(11) $${\left({\hat{K}}_{Y}\right)}_{j}=\sum_{i=1}^{n}{\left(s_{Y}\right)}_{ji}\cdot {\left(K_{X}\right)}_{i}.$$

In addition, based on the significant uncertainty of each keypoint, the weight of each keypoint can be calculated as

(12) $${\left(w_{Y}\right)}_{j}^{\prime }=\frac{{\left(w_{Y}\right)}_{j}}{\sum_{j=1}^{n}{\left(w_{Y}\right)}_{j}/n\;},\;\;$$

where ${\left(w_{Y}\right)}_{j}=\max\left\{\sigma_{max}-{\left(\sigma_{Y}\right)}_{i},0\right\}$, and $\sigma_{max}$ is a pre-set significant uncertainty maximum.

Above is the process of calculating the source point cloud keypoints and weights corresponding to each target point cloud keypoint; similarly, the target point cloud keypoints and weights corresponding to each source point cloud keypoint can be calculated. The final matching loss can be expressed as

(13) $$L_{matching}=\sum_{i=1}^{n}{\left(w_{X}\right)}_{i}\prime {\left\|R{\left(K_{X}\right)}_{i}+t-{\left({\hat{K}}_{X}\right)}_{i}\right\|}_{2}^{2}+\sum_{j=1}^{n}{\left(w_{Y}\right)}_{j}\prime {\left\|R{\left(K_{Y}\right)}_{j}+t-{\left({\hat{K}}_{Y}\right)}_{j}\right\|}_{2}^{2}.\;$$

## 3. Experiment

In this paper, the proposed point cloud registration framework is fully experimented on a large-scale point cloud dataset of key train components, and the results are accurately analyzed to verify the effectiveness and accuracy of the framework and to demonstrate the performance of the application on a large-scale point cloud of key train components.

### 3.1. Experiment Setting

The experiments were all implemented on a Windows 10 64-bit operating system based on the PyTorch 2.4.0 development environment and Python 3.9 programming language. Network training was performed on a desktop computer equipped with an NVIDIA GTX 3090 GPU using Adam as the optimiser with a learning rate of 0.001. Algorithm testing was performed on a desktop computer equipped with an NVIDIA GTX 3060 GPU. We trained the dataset for 2000 epochs. In a validity test, we trained two models with the dataset we constructed. For the DeepBBS network [36], we followed the original article to train the model using the Adam optimizer for 500 epochs with a batch size of two. The initial learning rate was set to 0.001 and was decreased by 10 after 260, 400, and 450 epochs. For the RORNet network [24], we followed the original article to train the model using a Radam optimizer for 500 epochs with a batch size of eight, and the learning rate was 0.001. We used the point-to-point ICP algorithm, and the threshold was 1 [1]. The maximum number of iterations was set to 50, and we did not perform more iterations in order to satisfy the requirement of registration time in practical applications, and the ICP algorithm tended to terminate early before the maximum iterations.

### 3.2. Dataset

In order to verify the effectiveness and applicability of the framework in this paper for large-scale point cloud registration tasks, we constructed a dataset using large-scale point clouds of key train components. The train key component data used are all large-scale point clouds with a 1–2 million point cloud volume, containing only the XYZ 3D coordinate values of the point cloud, and the file format was txt.

**Figure 5:**
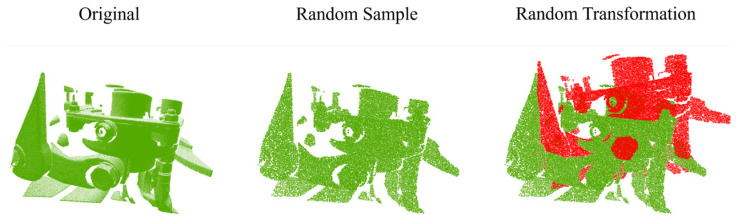
Example of constructing a train set. The original image was randomly sampled to 32,768 points and then randomly translated and rotated to obtain the registration point cloud pairs.

The training set was constructed using 568 data, and the components involved were mainly gearboxes and brake cylinders. Figure 5 shows the construction process of the training set. The 568 data were randomly sampled before training, all of which were sampled to 32,768 points, which were used as inputs to the network. The self-supervised approach was still used to randomly generate transformations during network training to obtain the corresponding source and target point cloud pairs, and the transformation matrix formed by rotating and translating between them was used as the real value of the two point clouds.

Three different datasets of key train components were produced for testing according to different experimental purposes, including the validity verification test set, same-scene application test set, and different-scene application test set.

**Figure 6:**
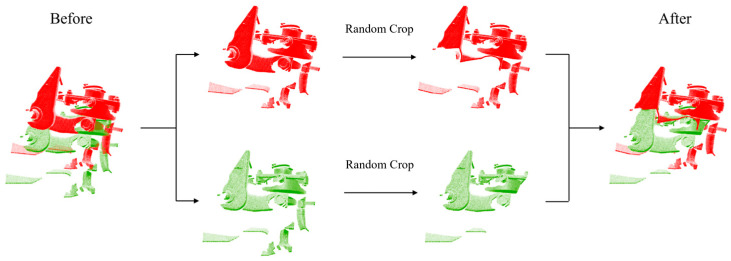
Example of constructing a partially overlapping dataset. A partially overlapping dataset was obtained by randomly cropping 80% of the points in the point cloud pairs based on the complete dataset.

When constructing the validation test set, the point cloud data of the gearbox and brake cylinder, which were different from the training data, were used. Like the train set, the original large-scale point cloud was randomly rotated and translated to obtain another point cloud, and the test set had a total of 178 data. Based on this complete dataset, part of the overlapping data in some of the registration experiments were obtained by randomly cropping two pieces of point clouds in each pair to retain 80% of the point cloud volume. Figure 6 shows a set of data processing.

**Figure 7:**
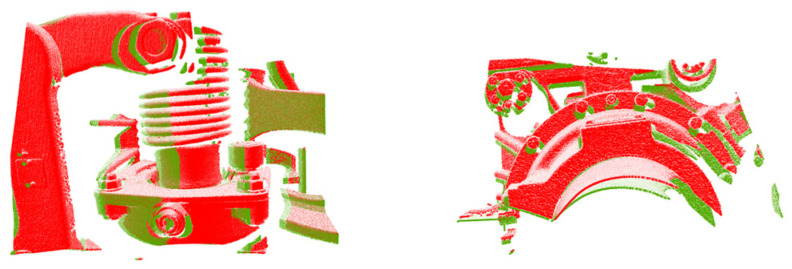
Example of constructing an application test set for the same scenario. Point clouds at different moments in the same scenario were obtained by laser scanning.

**Figure 8:**
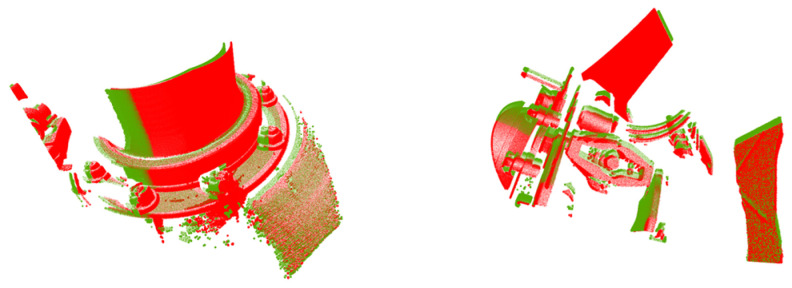
Example of constructing an application test set for different scenarios. Point clouds at different moments in different scenarios were obtained by laser scanning.

In practical applications, it is necessary to match two pieces of the point clouds of key train components at the same point collected at different times. Since the unknown and shape differences between two pieces of point clouds are not significant, the test directly took two pieces of large-scale point clouds as inputs to be processed by this network to obtain the results. When constructing the same-scene application test set, the data pairs of the gearbox and brake cylinder with the same scenes and similar point cloud shapes as the training data were used, as shown in Figure 7, with a total of 153 sets. When constructing the test set for different scene applications, data pairs with different scenes and different point cloud shapes from the training data were used, which came from a variety of key train components in a carriage collected at different times; two examples are given in Figure 8, which show that these scenes were significantly different from the training data, and there were a total of 152 groups in this test set.

### 3.3. Evaluation Metrics

When the transformation matrix between the source and target point clouds is known, the deviation between the predicted and true values can be measured by the commonly used evaluation metrics root mean square error (RMSE) and mean absolute error (MAE) to assess the goodness of the registration results. In this case, RMSE(R) and RMSE(t) are calculated as the average of the square root errors of the true and predicted values of the rotation matrix and translation vector, respectively, and MAE(R) and MAE(t) are calculated as the average of the absolute errors of the true and predicted values of the rotation matrix and translation vector, respectively, and the metrics are expressed as

(14) $$RMSE\left(R\right)=\sqrt{\frac{1}{n}\sum_{i=1}^{n}{\left(R_{pre}-R_{gt}\right)}^{2}},$$

(15) $$RMSE\left(t\right)=\sqrt{\frac{1}{n}\sum_{i=1}^{n}{\left(t_{pre}-t_{gt}\right)}^{2}},$$

(16) $$MAE\left(R\right)=\frac{1}{n}\sum_{i=1}^{n}\left|R_{pre}-R_{gt}\right|,$$

(17) $$MAE\left(t\right)=\frac{1}{n}\sum_{i=1}^{n}\left|t_{pre}-t_{gt}\right|,$$

where $R_{pre}$ and $t_{pre}$ are the prediction values of rotation matrix and translation vector, and $R_{gt}$ and $t_{gt}$ are the real values of rotation matrix and translation vector.

In addition, the rotation and translation matrix between point clouds is often unknown in practical applications, and without real values to assist in measuring the registration accuracy, the RMSE and MAE are no longer applicable. Therefore, in the application test of real train data, inspired by the nearest neighbor method to find the corresponding points and the literature [37], this paper, in addition to using visual observation as a subjective assessment method, also designed a distance error indicator $error(P_{pre},P_{target})$ to compare and measure the registration accuracy, which is calculated as the average of the Euclidean distances between the predicted point cloud $P_{pre}$ and the target point cloud $P_{target}$ with multiple sets of corresponding points, and can be expressed as

(18) $$error\left(P_{pre},P_{target}\right)=\frac{1}{M}\sum_{m=1}^{M}d_{m}=\frac{1}{M}\sum_{m=1}^{M}\left|{\left(P_{pre}\right)}_{m}-{\left(P_{target}\right)}_{m}\right|$$

Since the point cloud of the key components of the train used had millions of points, if the Euclidean distance was calculated for each point, there would be a lack of arithmetic power, so we selected a total of N pairs of corresponding points from the target point cloud and the source point cloud, and the corresponding points were selected in the following way: M points were randomly selected from the target point cloud, and M corresponding pairs of points were composed of 1 point in the collocation point cloud that had the smallest distance from the target point cloud through the nearest neighbor method. The average value of the distances of these M pairs of points was finally calculated as the distance error. In order to ensure the reliability of the results, when comparing the method of this paper with other methods, the same M points in the target point cloud were used to find the corresponding points in the alignment point cloud obtained by different methods, and then the distance errors were compared.

### 3.4. Results

#### 3.4.1. Sampling

Table 1 compares the time consumption of three commonly used sampling methods, voxel sampling, farthest point sampling, and random sampling, and it can be seen that as the number of sampling points and input points increase, the time consumptions of voxel sampling and farthest point sampling becomes longer and longer, which are far more than that of random sampling. Under the same conditions, random sampling has a faster processing speed and is therefore more suitable for solving the problem of sampling large-scale point clouds.

#### 3.4.2. Validity Test

**Figure 9:**
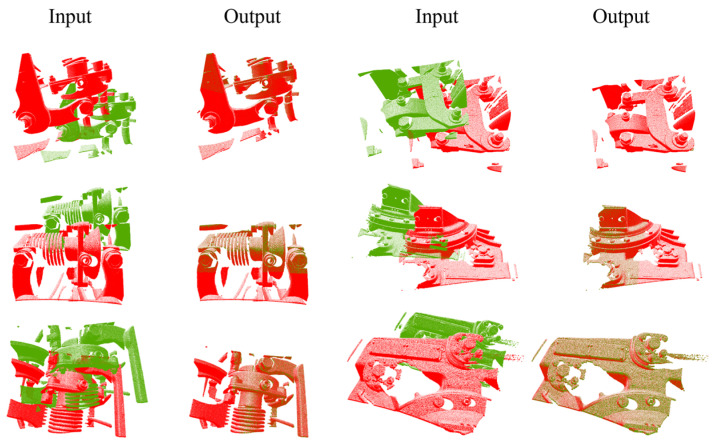
Point cloud registration results of our method on key train components.

The validity test matched the data in both the complete and partially overlapping cases, and the evaluation metrics were RMSE and MAE. Table 2 shows the registration results of the complete test set. This paper compares the effectiveness of several methods, including DeepBBS and RORNet networks. Using this paper’s method can effectively deal with large-scale point clouds, while the other methods, although they can show good results on small-scale point clouds, are difficult to be applied on large-scale point clouds, and many methods are almost ineffective. Figure 9 shows the visualization results of some key train components.

In order to be closer to the situation of incomplete point clouds in real applications, we also performed the effectiveness verification of partial alignment. The effectiveness test of partial registration is shown in Table 3. Compared to other methods, the method in this paper has a very good index and shows a high registration accuracy and robustness for partially overlapping large-scale point clouds.

#### 3.4.3. Application Test

Registration accuracy and computational efficiency are key indicators in practical applications, so we will consider registration accuracy and efficiency together when examining each method. Since the rotation translation matrix between two point clouds is unknown, the measure of registration accuracy combines the subjective assessment of vision and the objective assessment of distance error, and the registration efficiency calculates the time of the whole processing flow from the input of the large-scale point cloud to the output of the registration result.

In order to demonstrate the feasibility and advantages of the method in applications, we compared the registration results of using only the ICP registration algorithm, a Halcon engineering approach [38], only a coarse registration network based on keypoints and descriptors, and the registration method in this paper in two application test sets. The use of the coarse registration network refers to the registration results obtained from Figure 2, the green part of the network, and the RANSAC prediction of the transformed matrix.

**Figure 10:**
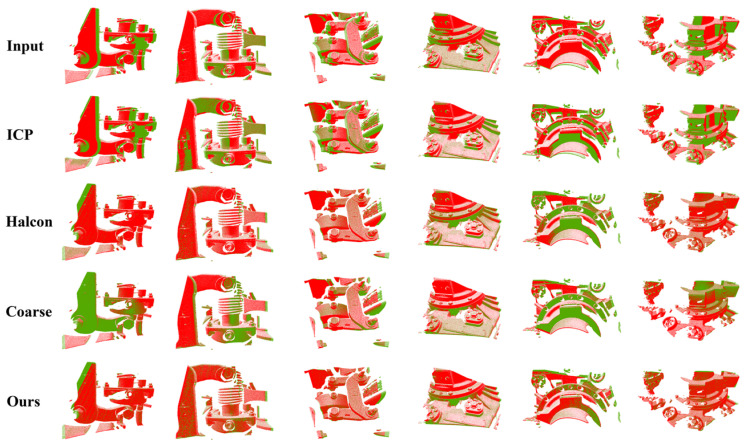
Application test visualization results in the same scenario.

Figure 10 show the results of the registration application in the same scenario, which are, from top to bottom, the results of the input point cloud pairs, the results of the registration using only the ICP method, the results of the registration using the Halcon approach, the results of the registration using the coarse registration network, and the results of the registration method in this paper. Combined with Table 4, it can be seen that, although the difference in the position of the two input point clouds is not large, due to the difference in the distribution of points, the number of points, etc., the point-to-point correspondence is difficult to find, so only using the ICP is simply not possible to match. The characteristics of the Halcon method are high accuracy and stability, but it is less efficient. The registration speed of the deep learning method is fast, which is much faster than the Halcon method, as shown in Table 4. However, its registration accuracy is slightly unstable, and the distance error is slightly larger than that of our proposed framework, but the difference is not significant. It can roughly match the positions of the two point clouds, which is convenient for the ICP to carry out fine registration. Compared with Halcon’s engineering method, the registration accuracy of this method is similar to that of Halcon’s, and both methods can achieve the purpose of accurate registration, but from the point of view of efficiency, our method can basically control the time of matching a pair of point clouds to less than 1 s, which reduces the average registration time by more than 10 times, and it can satisfy real-time requirements in engineering applications and greatly improves the processing efficiency.

**Figure 11:**
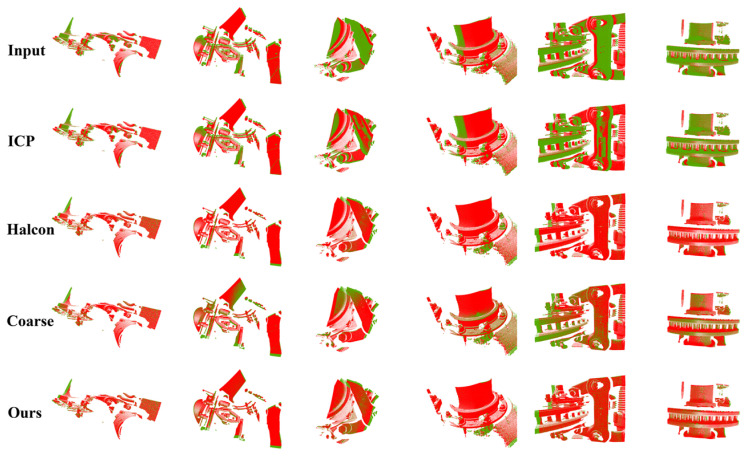
Application test visualization results in different scenarios.

Trains have a wide variety of components, some of which may or may not be similar to the data scenarios used for training, or even have completely different point cloud shapes. When training the networks, it is obviously not feasible to add the data of all the key components into the training set, which will consume a lot of training time, and it is also difficult to collect all types of data at one time, so it is usually used to use some of the scene data as a representative of the construction of the training set to participate in the training. In order to check whether the model trained by some scene data can complete the point cloud registration in more unknown scenes, we conducted the application test in different scenes, and the results are shown in Table 5 and Figure 11. Compared to the same-scene test, there is no change in the registration time, and the registration accuracy is slightly decreased, but it can still achieve a better registration effect, and it is still better than other methods, which indicates that the method has a certain degree of generalization, which is important for practical applications.

**Figure 12:**
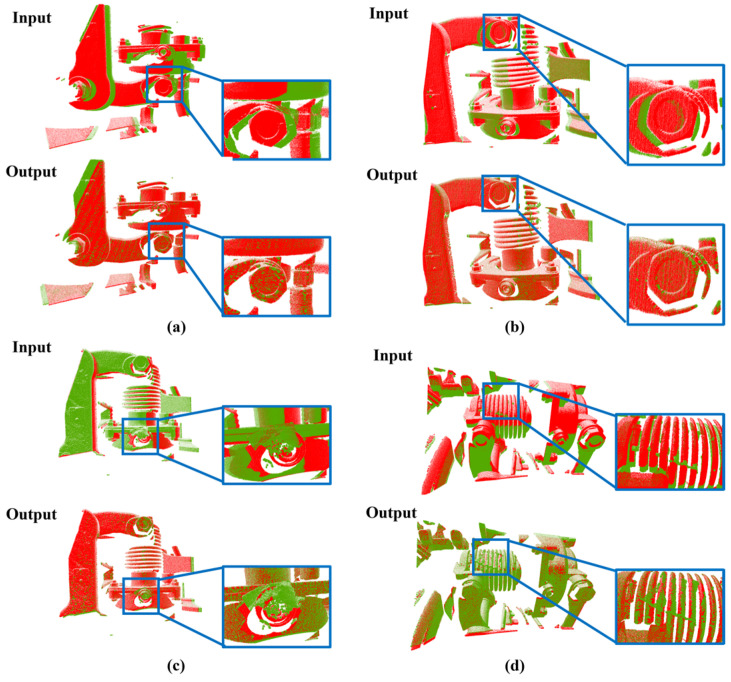
Example of registration of anomalous point clouds with normal point clouds. (**a**,**b**) are normal components after registration; (**c**,**d**) are abnormal components after registration.

In addition to the test metrics, the results can also be judged by direct observation with the human eye. In practical applications, two pieces of point clouds are effectively matched after registration, in which key parts such as bolts are matched accordingly. When such critical parts as bolts are missing, loose, covered, or exhibit other anomalies, and such a point cloud is compared to a point cloud with no anomalies under the same target, the anomaly can be clearly detected by direct observation with the human eye. Figure 12 demonstrates the results of the four sets of point cloud registration with zoomed-in local regions of the point clouds. In the figure, (a) and (b) show the registration between two normal point cloud pairs, and (c) and (d) show the registration between two point clouds with occlusion anomalies and the normal point clouds. It can be seen that the two point clouds overlap well overall after the registration. Observing the local area again, when there is no anomaly, the parts such as bolts in the local area can also be accurately matched. However, if there are anomalies, such as the zoomed-in positions in (c) and (d) in the figure, they do not overlap well after registration, indicating that there are obvious differences between the two point clouds, which provides help for the subsequent specific identification and detection of differences and defects in the condition.

## 4. Conclusions

Aiming at existing point cloud registration algorithms that make it difficult to solve the problem of large-scale point cloud registration, this paper proposes an efficient and stable point cloud registration framework, which makes use of random sampling to carry out simple and efficient downsampling processing for large-scale point clouds. It uses deep learning to construct a registration network based on keypoints and descriptors to accurately extract the feature point information of the point cloud and then completes the first registration by using the RANSAC and combines the ICP algorithm that has the advantage of accuracy to complete the second registration to form a stable registration framework. In addition, in the case that the rotation translation matrix of the point cloud is unknown, a distance error index is designed to measure the registration effect of each algorithm. Experiments on large-scale point clouds of key train components demonstrate the effectiveness of the registration framework. The method was applied to actual large-scale point clouds of key train components obtained from scans at different times, which show better registration accuracy and efficiency than many methods, proving the excellent performance of the method. Future work will be dedicated to solving the problems of large-scale point cloud registration with insignificant features and long model training times.

## Figures and Tables

**Table 1:** Time consumed by each sampling method under the influence of different orders of magnitude of input points and different numbers of sampling points.

Input Points Order of Magnitude	Sampling Point	Voxel Sampling	Furthest Point Sampling	Random Sampling
10^4^	1024	0.74 s	0.42 s	0.001 s
	2048	0.75 s	0.84 s	0.001 s
10^5^	16,384	1.19 s	6.87 s	0.002 s
	32,768	1.36 s	13.92 s	0.002 s
10^6^	16,384	3.95 s	13.71 s	0.03 s
	32,768	4.15 s	26.75 s	0.03 s

**Table 2:** Validity testing of complete data and comparison of methods.

Methods	RMSE(R)	RMSE(t)	MAE(R)	MAE(t)
ICP	8.7594	44.9727	7.6044	38.9110
FPFH + RANSAC	15.1094	77.6722	13.1290	67.1060
DeepBBS	16.1822	42.1172	11.2798	37.9836
RORNet	11.9933	51.3765	9.0477	44.1126
Ours	0.0277	0.1603	0.0244	0.1276

**Table 3:** Validity tests for partial registration.

Methods	RMSE(R)	RMSE(t)	MAE(R)	MAE(t)
ICP	9.8973	50.6800	8.6202	44.0030
FPFH + RANSAC	15.2712	76.0779	13.2904	65.6900
Ours	0.0650	0.4129	0.0568	0.3285

**Table 4:** Application test results in the same scenario.

Methods	Time/s	Error/mm
ICP	0.472	1.196
Halcon	9.349	0.395
Corse network	0.467	0.592
Ours (coarse-to-fine)	0.652	0.404

**Table 5:** Application test results in different scenarios.

Methods	Time/s	Error/mm
ICP	0.479	1.070
Halcon	7.022	0.458
Corse network	0.403	0.898
Ours (coarse-to-fine)	0.651	0.588

## Data Availability

The datasets presented in this article are not readily available because of technical limitations. But if anyone wants the raw data, I’m willing to provide some simple samples for reference.
